# A Hidden Markov Model for Analysis of Frontline Veterinary Data for Emerging Zoonotic Disease Surveillance

**DOI:** 10.1371/journal.pone.0024833

**Published:** 2011-09-16

**Authors:** Colin Robertson, Kate Sawford, Walimunige S. N. Gunawardana, Trisalyn A. Nelson, Farouk Nathoo, Craig Stephen

**Affiliations:** 1 Department of Geography and Environmental Studies, Wilfrid Laurier University, Waterloo, Ontario, Canada; 2 Spatial Pattern Analysis and Research Laboratory, Department of Geography, University of Victoria, Victoria, British Columbia, Canada; 3 Faculty of Veterinary Medicine, University of Calgary, Calgary, Alberta, Canada; 4 Faculty of Veterinary Medicine and Animal Science, University of Peradeniya, Peradeniya, Central Province, Sri Lanka; 5 Department of Mathematics and Statistics, University of Victoria, Victoria, British Columbia, Canada; The Scripps Research Institute Scripps Florida, United States of America

## Abstract

Surveillance systems tracking health patterns in animals have potential for early warning of infectious disease in humans, yet there are many challenges that remain before this can be realized. Specifically, there remains the challenge of detecting early warning signals for diseases that are not known or are not part of routine surveillance for named diseases. This paper reports on the development of a hidden Markov model for analysis of frontline veterinary sentinel surveillance data from Sri Lanka. Field veterinarians collected data on syndromes and diagnoses using mobile phones. A model for submission patterns accounts for both sentinel-related and disease-related variability. Models for commonly reported cattle diagnoses were estimated separately. Region-specific weekly average prevalence was estimated for each diagnoses and partitioned into normal and abnormal periods. Visualization of state probabilities was used to indicate areas and times of unusual disease prevalence. The analysis suggests that hidden Markov modelling is a useful approach for surveillance datasets from novel populations and/or having little historical baselines.

## Introduction

Approximately 75 percent of emerging infectious diseases (EIDs) in people are estimated to have originated in animals (i.e., zoonoses) [1]–[2]. Strategies to limit the impact of zoonotic EIDs can be broadly categorized as intervention at one or more of three levels: (i) controlling infections in people; (ii) blocking transmission of pathogens from animals to people; and/or (iii) preventing or controlling disease in animals [3]. Despite significant effort and funds targeting the first strategy, the global public health community continues to be caught off guard by EIDs. It is now recognized that the third strategy, control of disease in animals, may hold considerable potential for prevention of zoonotic EIDs [4]. To achieve this strategy, early detection of disease in animals is critical.

Surveillance for EIDs is confronted with the challenge of tracking something that has not yet happened. This has lead to the development of methods to track indicators of emergence or outbreaks such as risk factor surveillance and syndromic surveillance [5]. Surveillance systems using novel (pre-diagnostic) data sources that track healthcare-seeking behaviour have become widespread in human health surveillance with an aim to detect both intentional (bioterrorist) and naturally-occurring infectious disease outbreaks. Data representing early stage disease-related behaviours (e.g., staying home from work – absenteeism data) may have predictive value and promote detection of disease at the earliest possible stage. However similar data is generally not available for animals. EID surveillance systems must rely on pre-diagnostic, syndromic, or clinical diagnoses to gather early warning signals. Syndromic surveillance for early outbreak detection often uses automated data collection and ongoing analysis for statistical signals to monitor patterns in health outcomes in near real-time to detect early signals of diseases outbreaks [6]–[7]. Analysis of conditions frequently seen by field veterinarians but rarely recorded or tracked can be thought of as similar to a syndromic surveillance approach, in that the data represent novel and unknown populations and may have early warning value for emerging diseases. The data presented in this study is from a system which recorded clinical diagnoses of field veterinarians [8]. This system was developed as a prototypical complementary system to national disease reporting in Sri Lanka.

One of the drawbacks of pre-diagnostic, syndromic and clinical diagnostic data sources is that they incur an increased chance of false alarms [9]. With pre-diagnostic data sources, the data do not represent actual cases of disease, but variables related to disease - such as over-the-counter pharmaceutical sales [10], web site queries [11], or ambulance dispatch records [12]. Such data sources exhibit non-disease-related variations that need to be adjusted for in order to establish an accurate baseline level of risk. Similarly, clinical diagnoses data exhibit unknown variations that relate to how the data are collected. In many instances, making these adjustments is straightforward. For example, day of the week effects – that is, higher rates on certain days of the week - are features of many types of surveillance data. These higher rates could contribute to an outbreak signal when really the factors driving the increase are unrelated to disease, such as the greater propensity for people to visit the doctor on Mondays as compared to Fridays. With veterinary sentinel data, variability may be dependent on the sentinels themselves rather than the disease process. Therefore, with new and poorly understood surveillance data sources, developing a detailed understanding of baseline patterns (i.e., normal variation) is essential prior to conducting statistical analysis for cluster or outbreak detection.

Public health is increasingly looking towards surveillance of changing disease patterns in animals to enhance prediction and understanding of where and when EIDs in humans are likely to occur. Prediction of pre-emergence changes in pathogen dynamics in animals may hold the greatest potential of early detection in humans, and is therefore a central goal of EID surveillance [13]. A major challenge however, is the collection of appropriate data on animal health/behaviour [14]. For livestock populations, veterinarians may serve as an important source of information. However, using veterinary clinical diagnoses instead of results from diagnostic laboratory tests, the traditional data source in animal health surveillance, carries similar inherent risks to novel data sources in human surveillance systems: false alarms and unknown baseline variations.

There have been rapid advances in the development of appropriate methods of analysis for surveillance data [5], [15]–[16]. The detection of clusters in time [17], space [18], and space-time [19]–[20] are now routine analysis run in many surveillance systems (e.g., Heffernan et al. [21]). The majority of methods for cluster detection can be classified as hypothesis tests that evaluate the risk of some disease or syndrome within a subset defined by space/time, against some expected value estimated to be the normal state of the process. An alternate class of methods focuses on estimation of the expected value using statistical models. A modelling approach can incorporate known demographic risk factors such as age and occupation, or environmental risks such as sources of pollution that affect disease outcomes. Models have been used widely in influenza surveillance to account for seasonal dynamics [22], as well as long-term trends in retrospective analysis of chronic diseases [23].

Hidden Markov models (HMM) have recently been developed for disease surveillance applications [24]–[29]. A Markov model can be used to examine the probability of transition from one state (e.g., normal variation) to another state (e.g., abnormal variation). In a hidden Markov modeling framework, the data are related to a discrete-valued unobserved Markov process, and the dynamics of this latent process are inferred from the observed data. In disease surveillance applications, it is typical to assume that the latent process is a first-order Markov chain, with the values or states of this chain relating to mixture components corresponding to separate distributions for the observed data. (e.g., counts from separately parameterized Poisson distributions). In health surveillance applications, these states can represent the overall condition of the target population such as ‘endemic’ and ‘epidemic’, or ‘normal’ and ‘flu season’. A transition probability matrix governs transitions between the states over time. An advantage of HMMs for surveillance is that historical data are not required to train the model. Inferences about each of the states can be learned directly from available data, and in a Bayesian setting, the prior distributions. This is an attractive feature for new surveillance systems with short durations that lack baseline data.

In the first application of HMMs to surveillance, Le Strat and Carrat [24] demonstrated a Poisson HMM for poliomyelitis that estimated weekly counts of cases at the national level as a mixture of two Poisson distributions. Recent examples of HMMs being used in disease surveillance include healthy and unhealthy states related to health services utilization from medical insurance data [28] and outbreak and non-outbreak states of influenza [25].

In this paper, we report on a study investigating baseline patterns in an animal-based infectious disease surveillance system in Sri Lanka [8]. Data were collected for a period of a year describing clinical diagnoses of cattle, buffalo and poultry, in 4 regions of Sri Lanka. Field veterinary surgeons employed by the Department of Animal Production and Health submitted surveys via mobile phone to a central database. As these data describe syndromes and diagnoses not formerly tracked in Sri Lanka, there are no validation data available. We employ a modelling approach to examine different features of the data using hidden Markov models [24]. The objectives of the current study were to determine the sources of variation in animal-based EID surveillance in Sri Lanka, establish baseline rates for overall surveys, and explore spatial and temporal variability in commonly reported cattle diseases.

## Methods

### Data Sources

**Figure 1:**
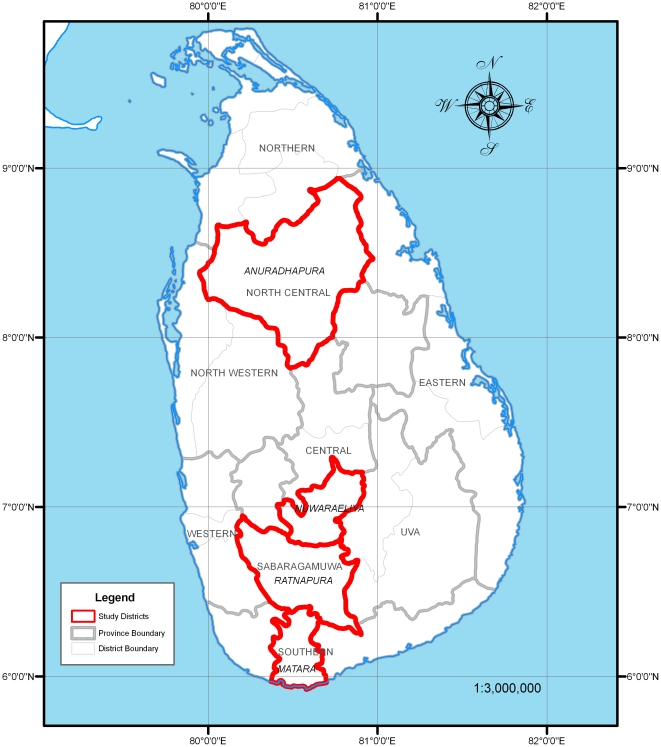
Study Area Map. Map of Sri Lanka and study districts that were part of the Infectious Disease Surveillance and Analysis System.

The Infectious Disease Surveillance and Analysis System (IDSAS) was established in January 2009 as part of a collaboration between the authors and the Department of Animal Production and Health in Sri Lanka [8]. The system tracked syndromes and clinical diagnoses in cattle, buffalo, and poultry, in four districts of Sri Lanka. Forty government-employed field veterinary surgeons (FVS) from four administrative districts (Figure 1) participated as data collectors using mobile phone-based surveys coupled with global positioning systems (GPS). FVSs were instructed to submit surveys via email to a central surveillance database for every encounter with one of the target species. The data used in the present study represent the period January 1^st^ 2009 to December 31, 2009, and the average monthly submission rate was approximately 11 surveys per month per FVS. All data obtained from farm and clinic visits made by veterinarians participating in the project remained the sole property of the Sri Lanka Department of Animal Production and Health and were used by the authors with full consent for research purposes.

Each survey submitted by a FVS represented one visit to a farm or one examination in clinic of at least one of the three species. Surveys were classified by routine visits (yes/no) and presence or absence of an animal health issue. In the case of an animal health issue, cases were given a syndrome group and a clinical diagnosis. FVSs also had the option of classifying the cause of the health issue as unknown. There were a total of 17 syndrome groups for cattle and buffalo and 11 for poultry. Options for suspected diagnoses were based on the syndromic grouping selected. For example, under “lameness”, possible diagnoses included Blackquarter, Footrot, Osteomyelitis, as well as 22 others. Each FVS was responsible for one geographic area called a range, so geographic locations could be associated with each survey. Farm-level spatial data collected with GPS were not used in this analysis, as we were primarily interested in determining broad-scale sources and patterns of variation in the IDSAS data.

Auxiliary data were collected to help account for non-disease variation in IDSAS data. FVS-specific information such as sex and the number of years since graduation from veterinary school was collected when the FVS was enrolled in the project. There were also specific dates when re-training was conducted and indicator variables were used to represent these periods. The retraining sessions increased enthusiasm and participation levels of the FVSs as sharp increases in submissions were noted in exploratory analysis of the data [8]. These factors represent what we term a *sentinel process*; factors related to the FVS as disease sentinels, rather than disease.

We obtained monthly temperature and precipitation data as district averages from the Sri Lankan Department of Meteorology as disease patterns in animals are often seasonal and may therefore exhibit a relationship with local weather patterns or seasons.

### Analysis of Surveillance Data

**Figure 2:**
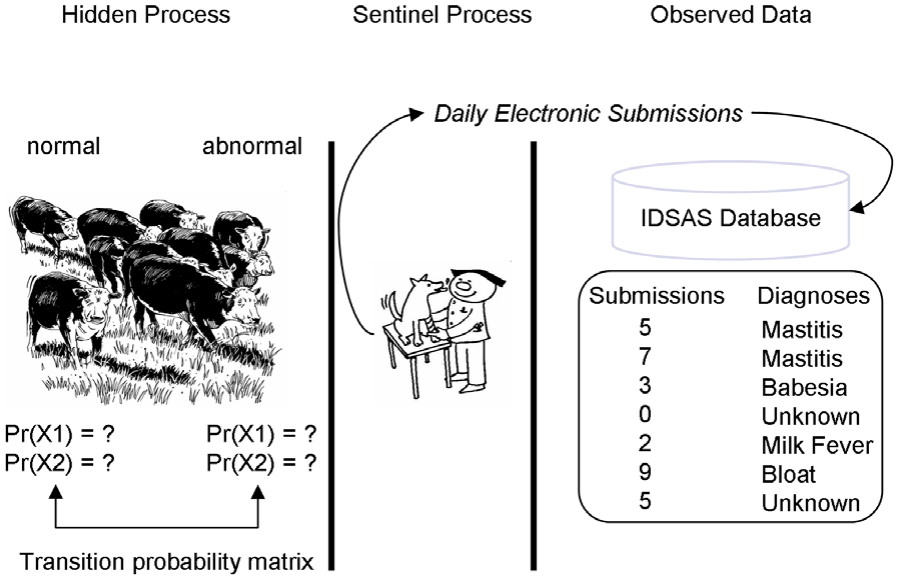
Data Generating Processes. Conceptual model of data generating processes in the Infectious Disease Surveillance and Analysis System in the context of hidden markov models. The hidden states of interest are the normal or abnormal state of animal health as seen by field veterinary surgeons. Observed data may include weekly submission counts, or counts of specific reported diagnoses.

In this study, we model animal health conditions as seen by FVSs in Sri Lanka. We extend on the spatial Poisson HMM for disease surveillance given in Watkins et al. [29], by simultaneously accounting for covariates (described above) impacting the observed data. The data collected by IDSAS can be conceptualized as arising from two independent processes, the *sentinel process*, and the *disease process* (Figure 2). We were interested in accounting for variability related to the sentinel process, in order to learn more about variability related to disease during the study.

To formulate our model, we let *Y_it_* denote the observed number of submissions to the IDSAS system during week *t* by FVS *i*. Underlying each observed count *Y_it_* is a latent variable S*_it_* taking one of two values, with S*_it_* = 1 corresponding to ‘normal’ conditions and S*_it_* = 2 corresponding to ‘abnormal’ conditions. We conceptualize ‘normal’ as the baseline and ‘abnormal’ as higher than baseline numbers of submissions. Conditioning on the latent state S*_it_* we assume the data are independently drawn from a Poisson distribution
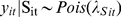
(1)with λ_1_ being the mean number of submissions in the normal state, and λ_2_ the mean in the abnormal state (i.e., λ_2_>λ_1_). The sequence of states occupied by the FVS *i* over time is represented through the vector ***S*** *_i_ = (S_1i,…_ S_Ti,_)′* and we assume each such sequence evolves from its initial state *S_1i_*, according to a first-order homogeneous Markov chain so that *Pr{S_it_|S_it-1_,S_it-2_,…,S_i1_} = Pr{S_it_|S_it-1_}*. The dynamics of this latent Markov model are governed by three unknown parameters: *P_Init_* an initial state probability governing the distribution of *S_1i_*, and two transition probabilities *P_12_* and *P_21_*, which represent the rates of transition between the normal and abnormal states. Following the parameterization in Watkins et al. [29], a Dirichlet prior distribution for initial probabilities, and Beta prior distributions on subsequent probabilities were employed. An outline of prior distributions for model parameters is given in Table 1. In what follows we shall denote this five parameter model (λ_1_, λ_2_, *P_Init_*, *P_12_*, *P_21_*) for total submissions as HMM_1_.

**Table 1:** Description of prior distributions and hyper-parameters for model parameters.

Model	Parameter	Prior Distribution	Description
HMM_1,2_	μ_1_	*Normal*(0,0.01)*	Mean state 1
HMM_1,2_	μ_2_	*Normal*(0,0.01)*	Mean state 2
HMM_1,2_	*P_Init_*	*Dirichlet*(0.5,0.5)	Initial Probability
HMM_1,2_	*P*	*Beta(0.5,0.5)*	Probability transition matrix
HMM_1,2_	*Y*	*Poisson*( 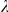 )	Observed count data
HMM_2_	*X*	*Normal*(0,0.001)*	Covariate coefficients

*Parameterized as mean and precision (1/variance, as in WinBUGS). For disease-level models, a *Normal*(0,10) prior was used to accommodate very small expected counts.

This model can be extended through the incorporation of covariates and this is typically done in one of two ways. First, we can allow the covariates to model variation in the Poisson parameters corresponding to the normal and abnormal states, where stationary between-state transition probabilities are assumed. Alternatively, covariates can be incorporated into an HMM via the transition probability matrix itself [30], resulting in an inhomogeneous HMM. For example, Wall and Li [28] present a HMM for medical service utilization data where covariates relate to transitions between healthy and unhealthy states via a logistic regression. In the model here, the former approach is adopted, maintaining stationary transition probabilities. Covariates were included in the model by relating each Poisson mean to a state-dependent baseline rate *μ_Sit_*, and a vector of FVS (i.e. spatial) and time specific covariates *X*, via a log-link Poisson regression:
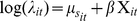
(2)where 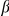 is the corresponding vector of regression coefficients which is assumed constant between the two states. The baseline rate, or intercept, is ‘switched’ between the normal and abnormal states based on the current state of the Markov chain.

The inclusion of covariates allows for spatial information to be included in the model. The four districts in which IDSAS operated were selected primarily to capture variation in environment, climate, and agricultural practices. For true outbreaks of disease or changes in pattern of disease, we might expect similar submissions among FVSs in the same district. To account for similarity of conditions within district versus other districts, submissions from FVSs in common districts were summed. The count *y_it_* of submissions for FVS *i* at time *t* was added to counts for all FVS in the same district.
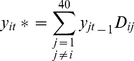
(3)where *D_ij_* is an *n*×*n* matrix with 1 s indicating FVSs in the same district and 0 otherwise. This information was included in a temporally lagged variable, representing the count of district wide submissions in the previous time period. We report results for the model with covariates included as HMM_2_.

All models were run on the individual submission counts to generate an understanding of the factors affecting the IDSAS data. To investigate the patterns of individual diseases, the four most frequently reported suspected diagnoses in cattle were investigated. Cattle are one of the primary livestock species assessed and treated by FVSs in Sri Lanka and as such constituted the majority of submissions. For the disease-specific models, covariate effects for sentinel-level variables were taken from estimates from the total submissions model, as we expect to these be constant factors effecting submissions equally. Disease-related variables (temperature, precipitation, and temporally lagged district-wide submissions) were estimated separately for each disease. Additionally, because natural disease prevalence varies by district, each district has separate mean rates for normal and abnormal states.

Models were implemented in a Bayesian setting with posterior distributions sampled using Markov chain Monte Carlo (MCMC) with implementation in WinBUGS [31]. Bayesian modelling is a convenient choice for developing HMMs as sensitivity to distributional assumptions can be easily assessed, and a full probability distribution is obtained for model parameters in the posterior distribution. In all analyses, two parallel MCMC chains were run for a 1000 iteration burn-in period followed by a production run of 4000 iterations. Convergence of the samplers to the corresponding stationary distributions was assessed using both visual inspection of the posterior sampling history, and the Gelman-Rubin statistic [32].

Model goodness-of-fit was evaluated using posterior predictive checking [33]. Simulated draws from the posterior distribution P(theta|Y) of model parameters were used to simulate replicate data sets Y^rep^ from the posterior predictive distribution P(Y^rep^|Y), which were used to compute the deviance (P[Y^rep^|theta]; computed as −2* log-likelihood) for each of 999 posterior and predictive draws. The deviance was then computed for the observed data, and the proportion of pairs (P[Y|theta], P[Y^rep^|theta]) where P[Y^rep^|theta]>P[Y|theta] is the posterior predictive p-value. Here, extreme p-values (i.e., 0.05>p>0.95 ) yield evidence of a poorly fitting model.

Results for the state variable are reported for two thresholds. The posterior mean state for each FVS/week pair (a total 2080) yield values ranging from 1.0 for ‘normal’ to 2.0 for ‘abnormal’ and values in between. We set a lower threshold of 1.50 to define membership in state two, and an upper threshold of 2.00. In all modelling results reported, coefficients with 95% credible intervals covering zero are excluded.

### Simulation Study

A simulation study was developed to evaluate model performance. Data from two Poisson models were simulated onto a 10×10 spatial grid representing disease-reporting units in a hypothetical surveillance system (n = 100). Three covariates were also simulated for each area. The normal state (i.e., state 1) Poisson model was as follows
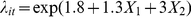
(4)and the abnormal state model (i.e., state 2) was
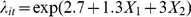
(5)Relationships for covariates *X*_1_ and *X*_2_ were the same between states but the intercept shifted from 1.8 during the normal state to 2.7 in the abnormal state. The purpose of the model is to detect shifts in state based on observations and simultaneously characterize the relationships between the mean and the covariate variables. We also evaluated whether the model could determine different covariate effects in different states, by changing the abnormal state model to include a third covariate:
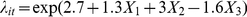
(6)In the simulation study analysis, spatial information (neighborhood relationships) was not used, but could easily be incorporated through a conditional autoregressive random effect, pooling observations from neighbouring areas, or including region-specific dummy variables.

**Figure 3:**
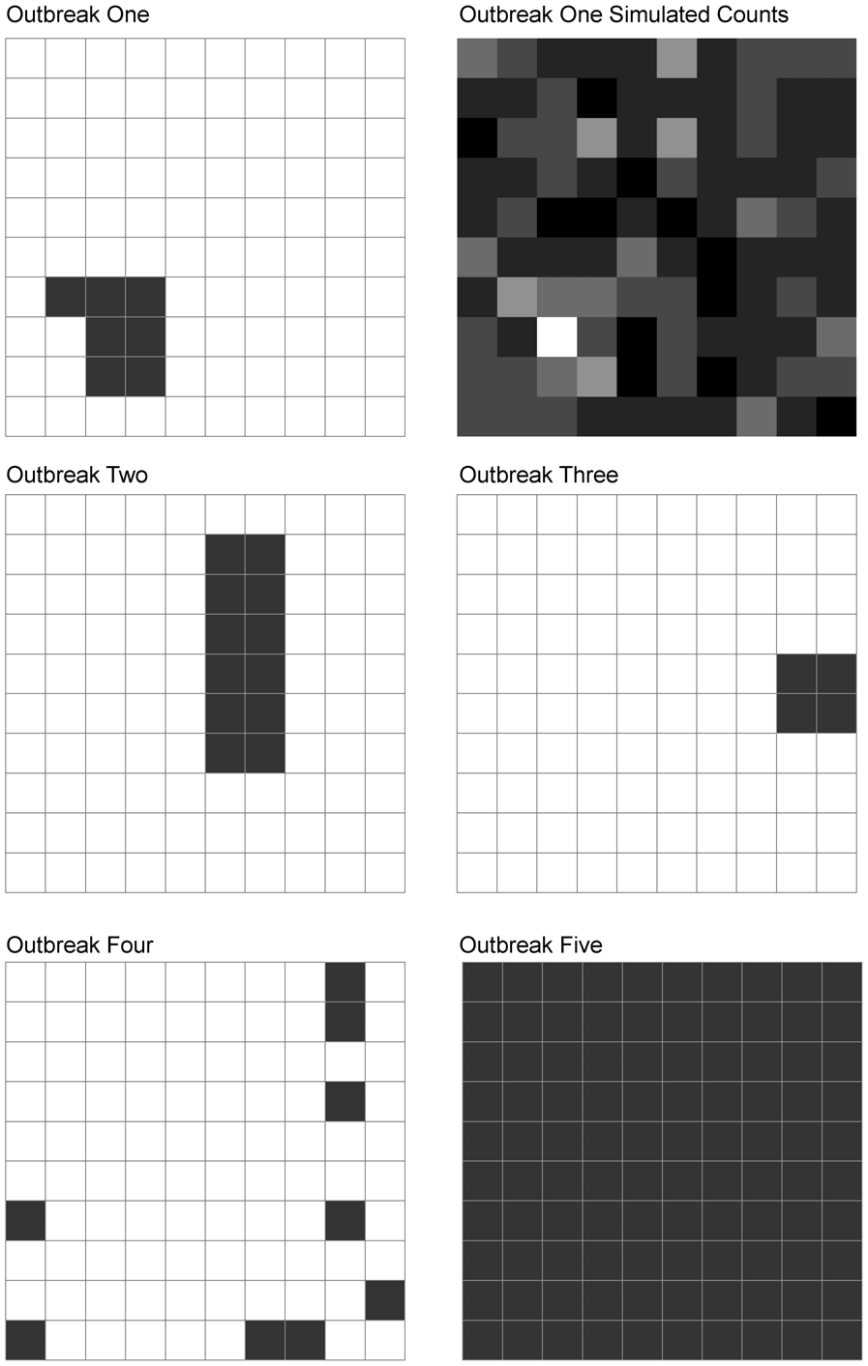
Simulated outbreak patterns in a hypothetical surveillance system. White cells generated under model for state one, and black cells generated under model for state two. The count data that was simulated using outbreak one is also shown: dark colours indicate low counts and lighter colours indicate high counts.

The normal state model was used to generate counts for 52 time periods (i.e., one year at weekly intervals) based on a normal distribution with a mean determined by Equation 4 and a standard deviation of 1. Different types of spatial patterns (outbreaks 1–5, see Figure 3) were created to establish areas where counts were replaced with counts estimated from the abnormal state model (Equation 5). Thus distinct spatial areas and time periods where counts and covariates in state two were created against a baseline of state one. In the second scenario, estimates for the abnormal state were obtained from Equation 6. Model performance was then evaluated as the percentage of correctly classified states.

## Results

### Simulation Study

The HMM model correctly classified 99.7% of the observations in the shifted intercept scenario. Out of 5100 (51 time periods×100 spatial units) observations (first week is not used because inference is based totally on initial values), 5088 were classified with the correct state. The 12 incorrectly classified states all occurred in outbreak five (see Figure 3), where all units were in the abnormal state, so all were errors of omission (i.e., incorrectly classified as normal). The coefficient estimates were similar to the true values for both variables, though the mean for the normal state was slightly underestimated (Table 2). In contrast, in the scenario with shifted mean and the addition of a third covariate effect in the abnormal state model, the model failed to converge completely. Posterior estimates for the intercept and covariate *X*1 were similar and converged (not reported), however estimates for coefficients on *X*2 and *X*3 both failed to converge. The model was run for 20,000 iterations. Convergence problems may be related to model identification issues, and these issues need further investigation, but are not uncommon with Bayesian mixture models employing weakly-informative priors.

**Table 2:** Model results from simulation study for five different outbreak scenarios occurring during a 52 week simulated surveillance system.

Parameter	True value	Posterior mean (95% credible interval)
μ_1_	1.80	1.73 (1.71, 1.74)
μ_2_	2.70	2.66 (2.63, 2.69)
*X1*	1.30	1.42 (1.29, 1.54)
*X2*	3.00	3.13 (3.03, 3.25)

### Animal Health Surveillance Submission Patterns

**Figure 4:**
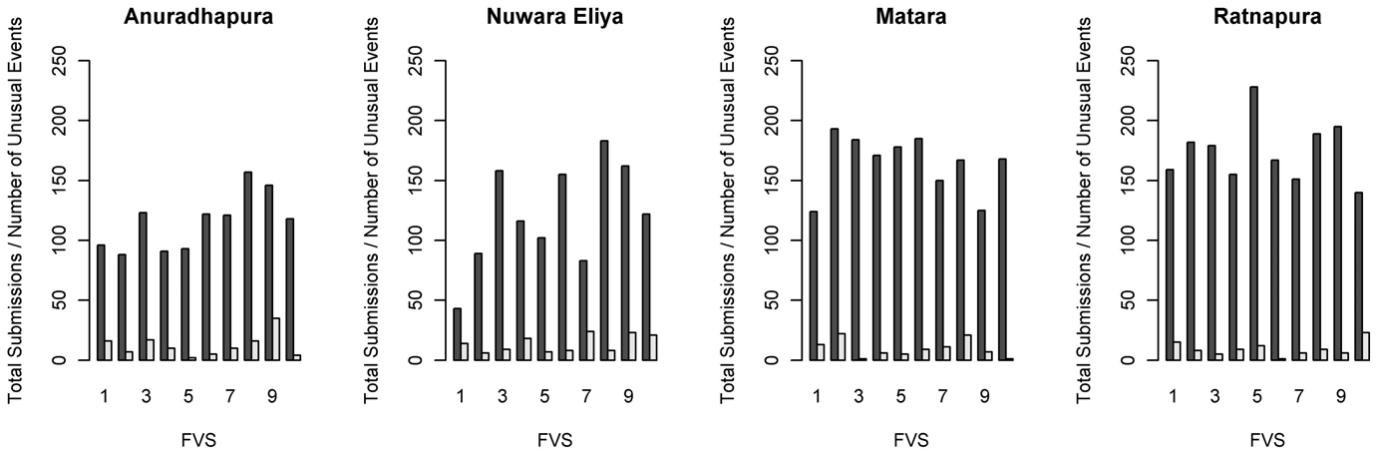
Submission counts and the number of unusual states per veterinarian. Total weekly submissions to the Infectious Disease Surveillance and Analysis System during the study period and the number of unusual states, by field veterinary surgeon and district. The number of weeks in state one (normal) is indicated in dark grey and the number of abnormal events in white.

**Figure 5:**
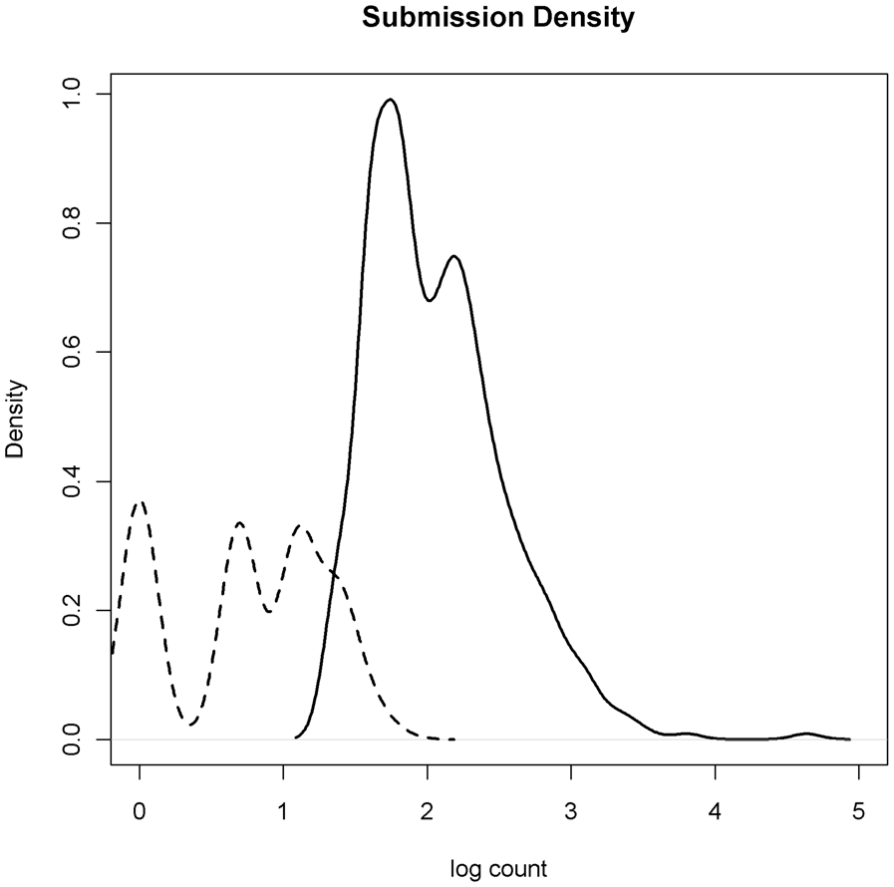
Submission count densities. Density of the log count of submissions in state one (dashed) and state two (solid).

During the study period, there were a total of 5758 submissions to the IDSAS system that reported an animal health issue. The HMM_1_ without covariates yielded a total of 753 abnormal events during the study period based on a posterior mean threshold of greater than 1.5. When constrained to a higher degree of certainty (posterior mean threshold of 2.00), the number of abnormal events was 390 (Table 3). The mean submission rate for state one was 0.45 (sd = 0.10) submissions per FVS, per week, and in abnormal periods the mean rate was 6.72 (sd = 0.05). When covariates were added to the model (HMM_2_), the number of abnormal events increased to 870 and 450 for the two threshold levels, while mean rates adjusted to 0.34 (sd = 0.10) and 6.65(sd = 0.10) submissions per FVS, per week for state one and two respectively. Covariate effects are reported in Table 3. Positive association with submission rates was limited to the variable indicating training periods, while covariates identifying male and less experienced FVS were negatively associated with submissions. Precipitation, temperature, and district reports had no effect in the total submissions model. The temporal patterns of abnormal events relative to all submission counts for each FVS are outlined in Figure 4. Using the upper threshold, the submission counts for state one ranged from zero to six, and from four to 103 for state two. The count densities plotted on a log scale are presented in Figure 5. Posterior predictive model checking did not reveal strong evidence indicating a lack of fit, with an overall posterior predictive p-value of 0.13 obtained for the deviance goodness-of-fit measure.

**Table 3:** Submission pattern model parameter estimates reported as rate ratios.

Parameter	Posterior mean (95% credible interval)	Standard deviation
μ_1_	0.34 (0.28–0.41)	0.10
μ_2_	6.65 (6.05–7.29)	0.05
Training	1.19 (1.08–1.32)	0.05
Years	0.59 (0.55–0.63)	0.03
Male	0.90 (0.84–0.96)	0.03

### Commonly Reported Cattle Diseases

**Figure 6:**
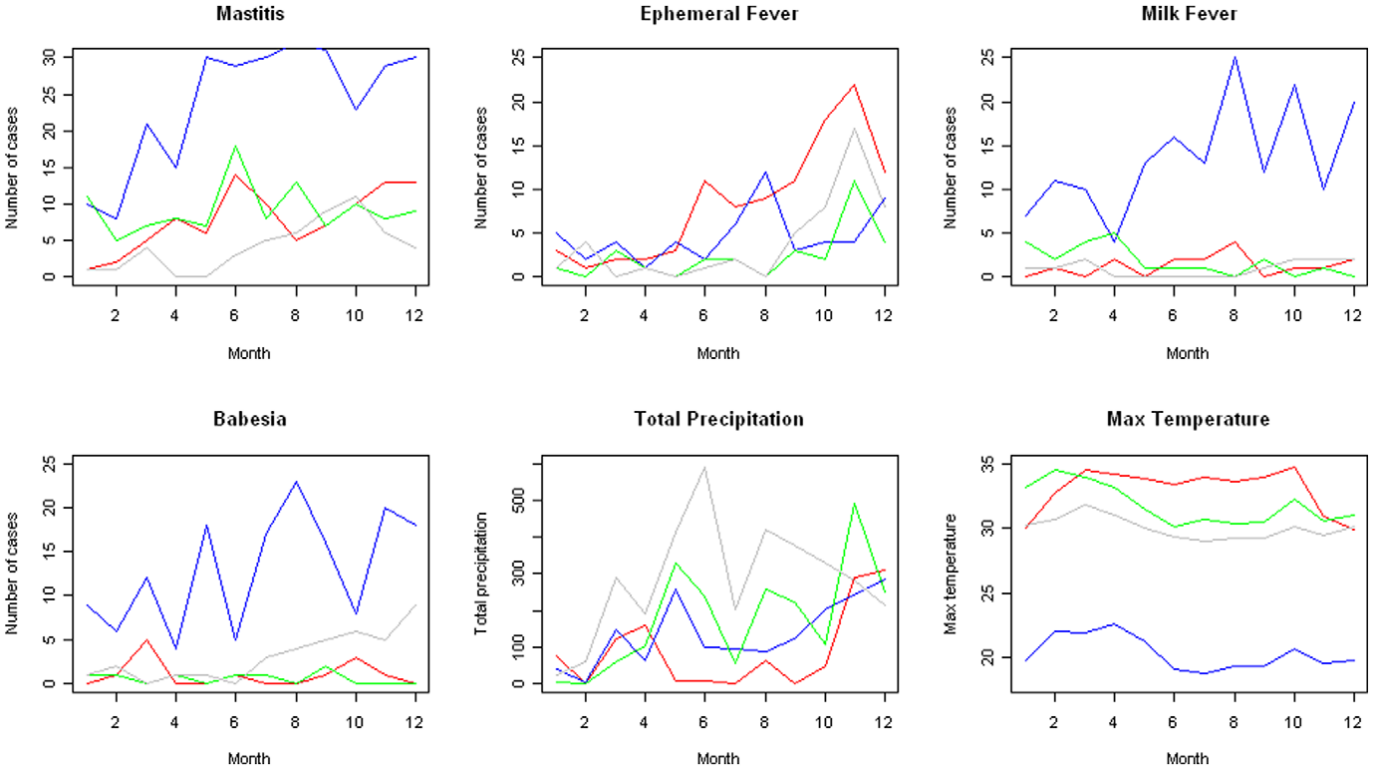
Monthly total cases for commonly reported diagnoses in each of the four districts. Anauradhapura (red), Nuwara Eliya (blue), Matara (green), and Ratnapura (grey). Monthly averages for district-wide total precipitation and maximum temperature.

In total, there were 3943 reported cattle cases during the study period. The most commonly reported diagnoses in cattle were mastitis (543), ephemeral fever (234), babesiosis (212), and milk fever (210). Monthly cases for each of the districts are given in Figure 6, along with environmental variables maximum temperature and total monthly precipitation.

Model results for the four most common diagnoses are outlined in Table 4. As noted earlier, coefficients for sentinel-level variables were set as estimated in the total-submission model, and only covariate effects for temperature, precipitation, and district reports were estimated for disease-level models. Overall, the effects of the covariate variables in disease-level models were minimal, with rate ratios ranging from 0.93 to 1.10. Temperature was positively associated with reported diagnoses of all diseases. Precipitation was not associated with diagnoses of any of the four diagnoses. Temporally lagged district reports were negatively associated with mastitis, babesiosis, and milk fever, and positively associated with ephemeral fever.

**Table 4:** Model results for four commonly reported cattle diagnoses.

	Anura-dhapura		Nuwara Eliya		Matara		Ratnapura		Temp-erature	Precipi-tation	District Reports
Model	μ_1_	μ_2_	μ_1_	μ_2_	μ_1_	μ_2_	μ_1_	μ_2_			
Mastitis	0.22	1.00	0.30	3.48	0.19	1.15	0.12	1.07	1.10	1.00	0.96
Ephemeral Fever	0.22	1.04	0.11	1.34	0.10	0.81	0.09	1.67	1.04	1.00	1.04
Babesiosis	0.09	0.79	0.24	3.51	0.08	0.63	0.13	1.05	1.10	1.00	0.93
Milk Fever	0.10	0.76	0.32	2.51	0.10	0.87	0.09	0.78	1.06	1.00	0.93

Posterior mean estimates are per week, per field veterinary surgeon, reported as rate ratios. Maximum daily temperature and total precipitation are computed for each district and month. District reports are the number of cases within the district in the previous week.

**Figure 7:**
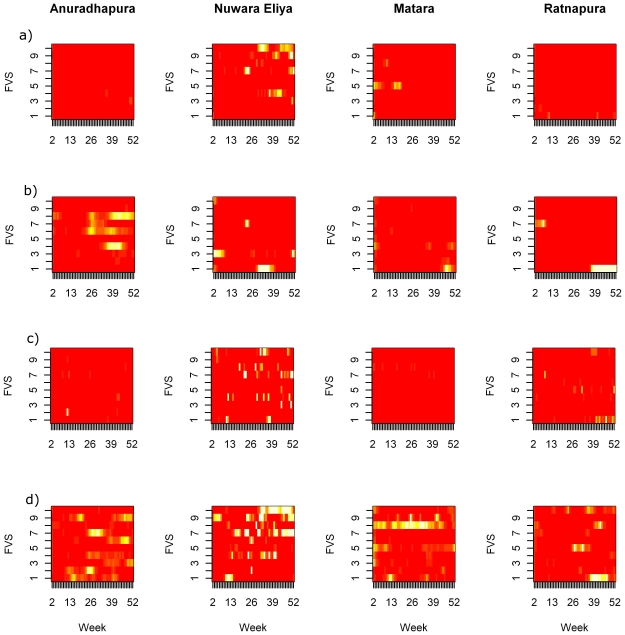
Posterior mean of the state variable. The model-adjusted posterior mean state for each field veterinarian surgeon by week, in each of the study districts for commonly reported cattle diagnoses. Red indicates state one and white indicates state two, and yellow intermediate values for a) Milk Fever, b) Ephemeral Fever, c) Babesiosis, and d) Mastits.

The posterior mean states are presented in Figure 7 for each of the four main disease categories. A possible outbreak of ephemeral fever is evident in Anuradhapura towards the end of the study period. Other periods of high submissions for babesiosis, milk fever, and mastitis are found in the Nuwara Eliya district.

## Discussion

Variation was modelled in data submitted to a mobile-phone based infectious disease surveillance system in Sri Lanka. Results indicate that submission varied according sentinel level factors, and that HMMs are a convenient methodology to approach novel sources of surveillance data. The average submission rate for surveys varied by district, ranging from 0.34 surveys per week during normal periods to in 6.34 surveys per week during abnormal periods. The number of abnormally high submissions increased when covariates were added to the model. Baseline estimates for normal patterns of mastitis, babesiosis, and milk fever were highest in Nuwara Eliya, the main cattle-dairy region in Sri Lanka. The baseline estimate for the normal pattern of ephemeral fever was highest in Anuradhapura, a region that experiences seasonal droughts.

The number of new pathogens in animals and humans are increasing and known infections are changing in pattern as natural and social systems adapt to changes in climate. The role of animals in emergence of new diseases is widely recognized [4], and surveillance of EIDs via animal-based systems such as IDSAS holds potential for detection and response at an early stage, yet studying this in the absence of an actual EID is a major challenge. While detecting an EID was the goal of the IDSAS system, enhanced understanding of the pathogen distribution as seen by veterinarians in the field represents an opportunity to both establish what is normal, and subsequently detect patterns that are unusual. This alone may be enough information to develop processes to inspire further action and promote early detection [34]. Further, the improved timeliness of IDSAS data as compared to laboratory testing is another attractive feature of using clinical diagnoses data for EID surveillance.

As this analysis has demonstrated, there are complex variations driving surveillance data using novel sources such as field-based veterinary surveys. In Sri Lanka, sentinel process factors such as the sex and work experience of the submitter impacted submission rates, as did periodic disruptions due to training and/or political events. The advantage of a modelling perspective to surveillance is that these sources of variation can be partitioned out in order to generate a finer understanding of the disease process. Previous analysis [8] using a subset of this data using the cumulative sum statistic on aggregated weekly submission counts, detected ‘outbreaks’ during the end of July and August (∼wk 30-31-38). In the model outputs here, it is evident that the high submissions during this period was confined largely to Nuwara Eliya. The model here provides greater geographical and temporal granularity while accounting for sentinel-specific non-outbreak variation. However, there is also value in learning about the sentinel process. This type of methodology could be used within ongoing surveillance systems to identify demographic characteristics more common amongst high submitters, and therefore serve to inform the sentinel selection process and ongoing sentinel inclusion or exclusion. In addition, exploration of the factors driving temporal variation in submissions can help to guide sentinel retraining and electronic prompts reminding sentinels to submit data.

When examining the results of the model HMM_1_ on total submissions, we note a high number of abnormal events. When variables are included in HMM_2_, the overall effect of the important variables actually reduces expected mean submissions, which results in more ‘unusual’ events. The question becomes, what is the value of accounting for sentinel-level factors. Given that alerts generated by surveillance systems typically overwhelm the number that can actually be investigated [35], should adjustments be biased downwards? The analysis here suggests that adjustments are useful because they provide a more complete understanding of the processes generating the surveillance data. In the context of sentinels for disease surveillance, this might simply be helping to identify characteristics that predict a more engaged sentinel relative to others. Another issue is that variables such as sex and experience may have an overall effect, but cannot be attributed to individuals. While states are discrete, state probabilities can be visualized across space and time as in Figure 7, providing visual evidence of gradual changes after covariates have been taken into account.

The simulation study presented here provides evidence that the model performs well under the scenario where a shift in the mean occurs and covariate effects remain fixed. In this simulation scenario, both the mean and covariate effects were recovered well by the model. This analysis lends support to the results obtained from IDSAS data. We might therefore be able to conclude overall, the means detected for each state-district combination in the disease-level models represent baseline estimates of the weekly prevalence of these diseases as seen by FVSs based on clinical diagnoses and syndromic groupings. However, the low values for these estimates (Table 4) make interpretation somewhat cumbersome. The state one means for all four diseases range from 0.09 for babesiosis in Anuradhapura to 0.32 for milk fever in Nuwara Eliya, while state two means ranged from 0.63 for babesiosis in Matara, to 3.51 for babesiosis in Nuwara Eliya. It is important to quantify the differences in means between the districts for the different diseases as it provides a starting point from which to understand why these differences exist. The trade off between data volume and data scale is characteristic of all statistical analysis and especially impacts analysis of surveillance data.

In developing this technique we chose to examine the four most frequently suspected diagnoses in cattle. However there are marked differences between babesiosis and mastitis in terms of epidemiology, etiology, and clinical presentation that are worth highlighting. Babesiosis is a tick-borne disease most commonly characterized by fever, inappetance, lethargy, weakness, red-tinged urine (hemoglobinuria), anemia and jaundice, though many cases are asymptomatic. Recovered cases become asymptomatic carriers, and duration of infection can be up to years. There is a large degree of variability in susceptibility between cattle breeds. Transmission of babesiosis is dependent on a bite from an infected Ixodes tick, and patterns in disease prevalence in cattle are dependent in part upon the prevalence of Babesia spp. in the vector and in the prevalence of the tick species itself [36]. In contrast, mastitis, defined simply as inflammation of the udder, can be caused by a variety of bacterial and fungal pathogens. It is often characterized by a drop in milk production, and when clinically evident may be accompanied by gross changes to milk or systemic illness. It can be caused by both contagious and environmental pathogens. Incidence and prevalence is impacted by a variety of individual animal characteristics, as well as environmental variables. Given these differences, it is worth considering whether examination of their occurrence using the same method is appropriate, and whether covariates should be fixed across suspected diagnoses.

Visualizing the probability of state two in Figure 7 on a FVS/weekly basis provides some evidence for the stability and confidence in the model inferences. The outbreak of ephemeral fever in Anuradhapura is on face value, more unusual, than for example patterns of mastitis in Nuwara Eliya. This is because based on what we know about ephemeral fever, transmitted by biting insects and often highly correlated with periods of rain, we expect, and are more concerned with ‘outbreaks’, than for mastitis, which is an endemic and pervasive condition. However, outbreak levels of mastitis may in fact represent clusters which also represent another, possibly unknown pathogen. The goal is to understand and establish the normal pattern for the population, so that unusual events can be quickly spotted and explored.

The reliability of inference in a models based framework will depend on the adequacy of the modeling assumptions, in practice, there are invariably important missing variables, and relationships often change over time in unforeseen ways. There are important limitations to the study that should be noted. Firstly, there exists the possibility of selection bias in our models. As we relied on farmers to report cases to veterinarians, perceived negative repercussions of reporting a severe or unusual disease could lead to underreporting of these types of cases by farmers. This would skew our data towards common and non-epidemic diseases. A second source of potential selection bias relates to the use of government veterinarians as data providers: while FVSs are significant animal health care providers, there are also private veterinary clinics in Sri Lanka, and commercial operations sometimes employ their own veterinarians. Cases assessed by private veterinary practitioners were not captured by the IDSAS system. Another limitation of the data is that biotic risk factors such as density dependence and interactions with wildlife were not tracked. These represent important drivers of zoonoses emergence. Going forward we hope to identify data sources that will help factor in these processes into our modelling approach.

Visualizing patterns of the state variable over time provides a quick diagnostic tool to identify changes in pattern. Also, because we are working within a Bayesian setting and have a full distribution for model parameters, we can make similar plots for the posterior uncertainty using posterior standard deviation. The modelling analysis here offers a robust framework for analysis of surveillance data with short temporal spans and multiple processes driving submissions, as is often the case with participant-generated data.
